# Ultrasound evaluation of varicoceles: systematic literature review and rationale of the ESUR-SPIWG Guidelines and Recommendations

**DOI:** 10.1007/s40477-020-00509-z

**Published:** 2020-07-27

**Authors:** Michele Bertolotto, Simon Freeman, Jonathan Richenberg, Jane Belfield, Vikram Dogra, Dean Y. Huang, Francesco Lotti, Karolina Markiet, Olivera Nikolic, Subramaniyan Ramanathan, Parvati Ramchandani, Laurence Rocher, Mustafa Secil, Paul S. Sidhu, Katarzyna Skrobisz, Michal Studniarek, Athina Tsili, Ahmet Tuncay Turgut, Pietro Pavlica, Lorenzo E. Derchi, Members of the ESUR-SPIWG WG, Jane Belfield, Mustafa Secil, Michele Bertolotto, Agnieszka Bianek-Bodzan, Irene Campo, Beniamino Corcioni, Lorenzo Derchi, Pieter De Visschere, Vikram Dogra, Simon Freeman, Caterina Gaudiano, Dean Huang, Oliwia Kozak, Francesco Lotti, Karolina Markiet, Olivera Nikolic, Alexandra Ntorkou, Raymond Oyen, Nicola Pavan, Pietro Pavlica, Thierry Puttemans, Subramaniyan Ramanathan, Parvati Ramchandani, Jonathan Richenberg, Laurence Rocher, Camilla Sachs, Paul Sidhu, Katarzyna Skrobisz, Michal Studniarek, Athina Tsili, Ahmet Turgut, Massimo Valentin

**Affiliations:** 1 Department of Radiology, University of Trieste, Ospedale Di Cattinara, Strada di Fiume 447, 34149 Trieste, Italy; 2 University Hospitals Plymouth NHS Trust, Derriford Hospital, Derriford Road, Crownhill, Plymouth, PL6 8DH Devon UK; 3 Brighton and Sussex University Hospitals NHS Trust, Royal Sussex County Hospital, Eastern Road, Brighton, BN2 5BE UK; 4 Royal Liverpool University Hospital, Prescot Street, Liverpool, L7 8XP UK; 5 Department of Radiology, University of Rochester, 601 Elmwood Ave, Rochester, NY 14642 USA; 6 Department of Radiology, King’s College Hospital NHS Foundation Trust, King’s College Hospital, Denmark Hill, London, SE5 9RS UK; 7 Andrology, Female Endocrinology and Gender Incongruence Unit, Department of Experimental and Clinical Biomedical Sciences, University of Florence, Viale Pieraccini 6, 50139 Florence, Italy; 8 Department of Radiology, Medical University of Gdansk, Mariana Smoluchowskiego 17, Gdańsk, Poland; 9 Faculty of Medicine, Center of Radiology, Clinical Center of Vojvodina, University of Novi Sad, Hajduk Veljkova 1-9, 21000 Novi Sad, Serbia; 10 Department of Clinical Imaging, Weill Cornell Medical College, Al-Wakra Hospital, Hamad Medical Corporation, PO Box 82228, Doha, Qatar; 11 Perelman School of Medicine, University of Pennsylvania, 3400 Spruce Street, Philadelphia, PA 19104 USA; 12 Department of Adult Diagnostic and Interventional Radiology, Bicêtre University Hospital, 78 Avenue du Général Leclerc, 94270 Le Kremlin Bicêtre, France; 13 Imagerie Par Résonance Magnétique Médicale Et Multi-Modalités, CNRS UMR8081, Service Hospitalier Frédéric Joliot, 4, Place du Gal Leclerc, 91401 Orsay Cedex, France; 14 Department of Radiology, Faculty of Medicine, Dokuz Eylul University, Izmir, Turkey; 15 Department of Clinical Radiology, Medical School, University of Ioannina, University Campus, 45110 Ioannina, Greece; 16 Department of Radiology, Faculty of Medicine, Istinye University, Istanbul, Turkey; 17 Private Hospital S. Maria Maddalena, Via Gorizia 2 – S. Maria Maddalena, 45030 Occhiobello, RO Italy; 18 Department of Health Sciences (DISSAL), Emergency Radiology, University of Genoa, Policlinico San Martino IST, Via A. Pastore 1, 16132 Genova, Italy

**Keywords:** Varicocele, Infertility, US, Doppler studies

## Abstract

Although often asymptomatic and detected incidentally, varicocele is a relatively common problem in patients who seek medical attention for infertility problems. Ultrasound (US) is the imaging modality of choice for evaluation, but there is no consensus on the diagnostic criteria, classification, and examination technique. In view of this uncertainty, the Scrotal and Penile Imaging Working Group of the European Society of Urogenital Radiology (ESUR-SPIWG) undertook a systematic review of the available literature on this topic, to use as the basis for evidence-based guidelines and recommendations. This paper provides the results of the systematic review on which guidelines were constructed.

## Introduction

Varicocele is defined as dilation of the pampiniform venous plexus draining the testicle, with reflux of venous blood [1, 2]. Although it can be asymptomatic and detected incidentally, it is a relatively common problem in patients who seek medical attention for infertility problems, or complain of chronic scrotal pain or discomfort [3].

Varicocele is detected and graded clinically using the criteria introduced by Dubin and Amelar in 1970, a subjective evaluation which is highly dependent on the expertise of the physician [4]. Colour Doppler ultrasound (US) is the imaging modality of choice [5], but the need for imaging itself is debated. In Europe, the use of US is recommended to confirm clinically suspected varicoceles, whilst in the USA and in Asia, routine use of imaging is not recommended [5]. Moreover, there is no agreement on how to perform the US examination. A variety of classifications is used based on different sonographic parameters, even within the same country, depending on the practice of the individual sonologist and of the referring clinician [2].

There is a large, but extremely heterogeneous body of published investigations on US imaging of varicoceles. Even though variability makes it impossible to perform a metanalysis, a systematic literature review is possible.

The Scrotal and Penile Imaging Working Group of the European Society of Urogenital Radiology (ESUR-SPIWG) attempted this task. The group performed a systematic review of the available literature and released a guideline and recommendation paper with the aim to standardize US imaging for varicoceles in Europe. After having formulated 15 relevant clinical questions (Table 1), 23 evidence-based recommendations are provided (Table 2) [6].

**Table 1:** Questions formulated by the ESUR-SPIWG to deal with the difficulties encountered in imaging varicoceles

Questions
Question 1: What is the evidence for correlation between varicocele, spermatogenesis damage and infertility?
Question 2: How are varicoceles classified by ultrasound?
Question 3: Should the size of the dilated veins be measured? Should measurements be performed standing or supine, at rest or during the Valsalva manoeuvre? Which size threshold should be used for the dilated veins?
Question 4: When to measure testicular size at ultrasound, and how?
Question 5: How should US be performed in patients with varicoceles?
Question 6: Is Doppler evaluation of venous reflux needed and which parameters should be measured?
Question 7: How long should reflux last to make the diagnosis of varicocele?
Question 8: Is reflux velocity clinically important, and how should it be measured?
Question 9: How should US examinations be reported in patients with varicoceles?
Question 10: Is evaluation of intra-testicular Doppler waveforms worthwhile in imaging varicoceles?
Question 11: Can evidence-based recommendations be provided for imaging right-sided varicoceles?
Question 12: Is imaging follow-up necessary for subclinical varicoceles?
Question 13: Should patients be followed-up after varicocele treatment?
Question 14: Is it always necessary to examine the abdomen for tumours in patients with a newly discovered varicocele?
Question 15: What are the pitfalls in US when imaging varicoceles?

Ultrasound evaluation of varicoceles: guidelines and recommendations of the European Society of Urogenital Radiology Scrotal and Penile Imaging Working Group (ESUR-SPIWG) for detection, classification, and grading (Ref. [1], reprinted with permission)

**Table 2:** Authorized recommendations from the ESUR-SPIWG for ultrasound evaluation of varicoceles

#	Recommendations	LoE	GoR
1	Grey scale and Doppler US modes are used to assess the parameters required for varicocele classification. There is no universally recognized classification system	3	C
2	Given the widespread methodological variability that exists in measurements of venous diameter in varicocele assessment, it is critically important to document the patient’s position, whether measurement was made at rest or during the Valsalva manoeuvre, and the location of the measured veins relative to the spermatic cord or testis	1	A
3	Measurement of the largest vein, irrespective of location, with the patient in the upright position and during the Valsalva manoeuvre is recommended	5	D
4	A maximum venous diameter of 3 mm or more can be considered diagnostic for a varicocele when measured with the patient in the upright position and during the Valsalva manoeuvre	2	B
5	Testicular volume should be measured in all cases as it correlates with testicular function in both infertile patients and patients with a varicocele	1	A
6	Accurate measurement of the three diameters of the testis is required to obtain testicular volume estimation. Use of Lambert’s formula (*V* = L × W × H × 0.71) is recommended. The mathematical formula used to calculate the volume should be reported	2	B
7	A standardised protocol is required for varicocele ultrasound examination. A grey-scale and colour Doppler examination, with spectral Doppler analysis, should be performed bilaterally with the patient supine and standing, during spontaneous breathing and during the Valsalva manoeuvre	2	B
8	Demonstrating and evaluating reflux flow in patients being assessed for varicoceles is the most important part of the Doppler ultrasound study	3	C
9	Colour Doppler interrogation should be supplemented with spectral Doppler analysis. Reflux duration is the essential parameter to be measured (LoE 3, GoR C). Measurement of the reflux peak velocity is optional	5	D
10	Reflux in the testicular veins lasting more than 2bs with the patient standing and during the Valsalva manoeuvre should be considered to be abnormal	4	C
11	There is insufficient data to recommend using reflux peak velocity measurements as a factor in determining the need for varicocele repair	5	C
12	When issuing reports on patients with varicoceles, the examination technique should be described	1	A
13	Grading varicoceles according to the Sarteschi’s classification may be helpful in clinical practice. For standardisation purposes, it is recommended that all the US parameters used to evaluate the patient are also reported	5	D
14	Evaluation of intra-testicular blood flow in patients with varicoceles is an active research field which might provide a valuable insight into the mechanisms that create testicular parenchymal damage. At present, however, this evaluation cannot be recommended for clinical use	3	C
15	Bilateral colour Doppler US should be performed in patients with left-sided varicoceles as it will frequently reveal subclinical right-sided varicoceles	3	B
16	In patients with an isolated clinical right-sided varicocele, US can be extended to the abdomen to look for abdominal and retroperitoneal pathology, as well as congenital vascular anomalies	5	D
17	In patients with subclinical varicoceles imaging, follow-up is recommended in all adolescents who have not undergone surgical repair and in young adults with normal semen analysis and normal testicular volume	3	C
18	After varicocele repair, US can be used to identify early postoperative complications	3	C
19	Sperm analysis forms the basis of follow-up following varicocele repair. The data available do not support the routine use of US	1	A
20	Colour Doppler US can be used after varicocele repair if semen analysis remains unsatisfactory to evaluate testicular volume and identify signs of persistent or recurrent disease	2	B
21	Extended US examination to the abdomen is recommended in children less than 9 years of age presenting with acute varicocele (LoE 2, GoR B)	2	B
22	There is insufficient evidence to conclude that an extension of the ultrasound examination of the abdomen is mandatory in all adult patients with a varicocele. The ultrasound practitioners should use their clinical judgement to decide whether to proceed to an abdominal examination, particularly if the varicocele is large, of recent onset and persists with the patient in the supine position	5	D
23	In patients being investigated for a clinically detected varicocele, the possibility of rare varicocele mimics should be considered. The correct diagnosis can usually be made by combining the grey-scale and Doppler US features	5	D

Source: Ultrasound evaluation of varicoceles: guidelines and recommendations of the European Society of Urogenital Radiology Scrotal and Penile Imaging Working Group (ESUR-SPIWG) for detection, classification, and grading (Ref. [1], reprinted with permission)

The systematic literature review at the root of these recommendations is presented in its complete form in this work.

The ESUR-SPIWG believes that standardization was necessary not only to improve clinical practice, but also to define the methodological basis for future studies and metanalyses, with the hope of a better understanding of the relationship between varicoceles and infertility.

## Methods

Data for this systematic review were collected according to the PRISMA statement 2009 guidelines [7]. Eligibility criteria and methods of analysis were specified in advance. Both controlled and non-controlled studies were included, as well as case–control and cohort studies. Search strategy selection and methods for data extraction and data analysis have been previously reported in detail [6].

## Limitations of the literature

ESUR-SPIWG proceeded with full awareness of the limitations of the varicocele literature. These limitations include heterogeneous patient groups, small sample sizes, lack of studies with diagnostic accuracy data, lack of randomized controlled trials or controlled studies with patient outcome data, and use of a variety of outcome measures. Overall, these difficulties precluded use of meta-analytical procedures. Instead, narrative syntheses were used to summarize the evidence for the questions of interest.

When review of the literature revealed insufficient publications to address a recommendation from an evidence basis, the recommendation was obtained from expert opinion based on clinical practice, experience, knowledge and judgment. If differences of opinion among the expert emerged, consensus was achieved using the modified Delphi technique [8].

## Systematic review to address question 1

Understanding whether the alleged correlation between impaired spermatogenesis, infertility and varicocele is evidence based is fundamental in making a recommendation for US as a valuable investigation in infertile patients. Infertility is defined as “failure to achieve a clinical pregnancy after 12 months or more of regular unprotected sexual intercourse” [9]. Approximately 15% of couples do not achieve a pregnancy within the first year, although almost half of them will do so in the second year. Up to 12% of men have fertility problems, and when a specific cause for infertility can be identified, a male contributing factor is found in approximately 45–50% of cases [5, 10]. Varicocele is the most commonly identifiable and treatable potential cause of male subfertility, with an estimated prevalence of 15% in the general population, 40% in sub-fertile men, and 75–81% in men with secondary infertility [11–13]. There is evidence that clinically palpable, rather than non-palpable, varicoceles are associated with infertility but a correlation between the degree of testicular tissue damage and the size of a varicocele is not proven [14].

Both clinical and laboratory studies show a detrimental effect of varicoceles on spermatogenesis [15, 16]. Testicular function is usually normal at the age of 18–20, but declines progressively depending on the duration of the varicocele [17]. A clinical varicocele is associated with ipsilateral testicular atrophy which may improve following varicocele repair [18–20].

Several mechanisms have been proposed to explain the link between varicoceles and impaired spermatogenesis. A multi-factorial aetiology is likely, involving both heat, oxidative stresses and androgen deprivation [21]. The temperature of the testes is usually 2 ℃ below core body temperature; if increased by a varicocele, this can lead to a reduction in sperm quality and increased sperm apoptosis. Testicular temperature reduces following varicocele repair [22–25]. Increased venous hydrostatic pressure may also result in testicular hypoxia by causing oxidative stress through the creation of reactive oxygen species which may reduce sperm quality through several different mechanisms including DNA damage and fragmentation [26, 27]. Varicocele repair has been shown to reduce the seminal oxidative stress in men with infertility [28, 29]. Adverse effects on Leydig cell function and decreased intra-testicular testosterone levels may also be additional contributing factors [21, 30]. Adrenal catecholamines reflux has also been considered among the factors responsible of fertility impairment in patients with varicoceles [31].

Although sperm quality will frequently improve following treatment, there is conflicting evidence regarding the value of varicocele repair in male infertility [32–34]. A systematic review in 2015 found that there was insufficient evidence to support a beneficial effect of varicocele repair on spontaneous pregnancy and only limited evidence that treatment might be beneficial in men with clinical varicocele and abnormal semen parameters [35]. Other studies reported potential beneficial effects in pregnancy outcomes following varicocele repair in sub-fertile men [32, 36, 37]. In the setting of assisted reproduction, another meta-analysis found that varicocele repair resulted in improved live birth and pregnancy rates with in vitro fertilization or intracytoplasmic sperm injection treatment [38]. Many of these studies have concluded that the current level of evidence is insufficient for providing a definitive opinion.

Guidelines for varicocele repair in subfertility are inconsistent. In the UK, the National Institute for Health and Clinical Excellence guidelines advises that surgery for varicocele should not be offered for fertility treatment as there is no definite evidence that it improves pregnancy rates. Opinion from the American Society for Reproductive Medicine and the Society for Male Reproduction and Urology concludes that varicocele repair may be offered to the male partner of an infertile couple where there is evidence of abnormal semen parameters and no/minimal identified female factor [39].

## Systematic review to address question 2

Evaluation of the diagnostic potential of US imaging in patients with varicoceles is markedly hampered by difficulties in comparing studies obtained with different US classifications and different imaging parameters. A consensus should play a key role for obtaining more reliable results in future studies.

There is general agreement in Europe that valuable information about varicocele can be obtained using colour flow Doppler, and that evaluation of the presence and characteristics of venous reflux is useful when physical examination alone is insufficient to determine whether treatment is required. Additionally, colour Doppler evaluation plays a key role in the assessment of persistence or recurrence of varicoceles after surgical treatment; veins often remain dilated and clinical examination alone cannot fully assess the presence or absence of persistent venous reflux.

Patients with reflux have significantly impaired semen analysis parameters compared to patients without reflux [40]. A prospective study shows that the main predictive factor of a better seminal response after varicocele correction is evidence of venous reflux at preoperative colour Doppler US [41].

Consequently, a number of sonographic classifications have been proposed to establish firm criteria for the diagnosis, treatment and prognosis of varicocele [2, 42–51]. Unfortunately, examination techniques differ for each of these classifications, and different parameters are used to categorize the severity of the disease. A strict comparison between data from different studies is, therefore, not applicable and pooling the available data to construct a metanalysis is problematic.

Some classifications grade varicoceles according to the characteristics of venous reflux only. The patient is evaluated either supine, upright, or in both positions. Hirsh et al. classify varicocele in three grades according to reflux duration while standing during the Valsalva manoeuvre. Cornud et al. classify varicoceles in three categories according to the duration of reflux during the Valsalva manoeuvre measured with spectral Doppler interrogation; there is no standardisation as to whether the examination should be performed with the patient supine or standing [47]. Oyen and Dabuwala et al. classify reflux into three grades based on the characteristics and length of reflux measured with the patient supine [43, 48]. Iosa and Lazzarini score varicoceles in five grades based on a qualitative evaluation of venous reflux estimated with patient both standing and supine [50]. Patil et al. redefined the criteria used to grade varicoceles with four scores based on reflux times measured while standing [51].

Other classifications use both morphological and Doppler parameters. Hoekstra and Witt score varicoceles by measuring the size of the dilated veins and the presence of reflux during the Valsalva manoeuvre in the upright position. Chiou et al. developed a system which assigns a maximum score of 9 points incorporating the maximum venous diameter (scores 0 to 3), the presence of venous plexuses (scores 0–3) and changes in flow direction during the Valsalva manoeuvre (scores 0–3). Diagnosis of varicocele requires a total score of at least 4 points [46]. All parameters are evaluated with the patient lying supine.

The Sarteschi’s classification, first published in Italian in 1993 [44] and published in English with minor changes by Liguori et al. in 2004 [52], distinguishes five degrees of varicocele, depending on the presence of dilated veins while supine and/or standing, anatomical relationships of the dilated veins with the testis, characteristics of reflux, and testicular size. Pauroso et al. introduced a simplified version of the Sarteschi’s classification based on both varicocele extent and the magnitude of the reflux; the two parameters were assessed only with the patient lying supine [49].

The purpose of every classification system is to provide useful morphological and hemodynamic information for treating varicoceles and predicting treatment outcomes. Unfortunately, a clear consensus has not been reached, and there is no universally recognized system to classify varicocele severity. The thresholds used to differentiate between normal and pathological findings are often different and even conflicting, resulting in US examinations that can be interpreted as positive or negative for varicocele diagnosis and severity depending on the classification system used. The result is that all grading systems have a low predictive value in terms of the effect of varicoceles on impairment of spermatogenesis as well as on sperm quality improvement after varicocele correction.

Since no classification system for varicoceles is universally recognized, the ESUR-SPIWG recommends recording all the parameters of the different classifications, to allow comparison among the results of different studies, regardless of which classification system is used (Table 2, recommendations 1).

## Systematic review to address question 3

It is uncertain which venous diameter should be regarded as abnormal for varicocele diagnosis. Additionally, the utility of measuring the dilated vein is debated, as well as where in the scrotum measurement should be obtained, in which patient’s position (standing or supine), and how (at rest or during the Valsalva manoeuvre). A standardised method to measure the veins is necessary to compare results obtained in different studies.

The size threshold of the veins that meets the definition for varicoceles varies in different studies, as does the examination technique employed. In particular, venous diameter is evaluated in the supine position or while standing, either at rest or during the Valsalva manoeuvre in different papers [45, 53–62]. Measurement sites of venous diameter in the scrotum are also variable. This is clinically relevant, because the size of the dilated veins changes with the patient’s position, with the Valsalva manoeuvre, and with the measurement site [57, 60, 62]. The result is a wide variation of measurements both in normal subjects, and in patients with clinical and subclinical varicocele.

**Fig.1:**
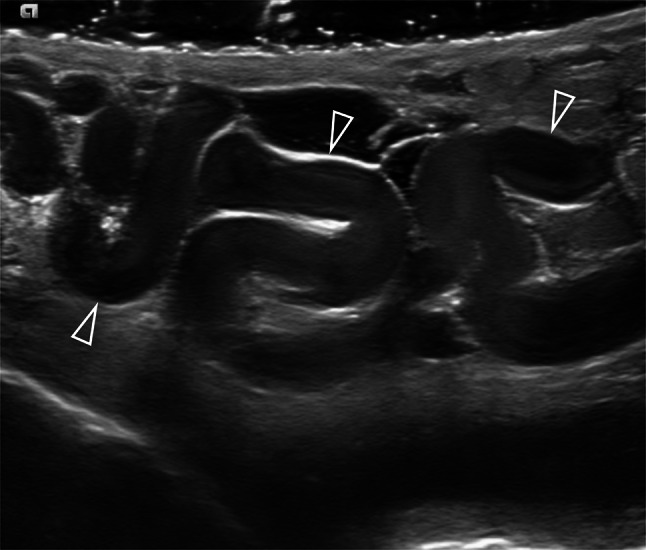
Grey-scale appearance of varicocele. Multiple, hypoechoic serpiginous dilated veins (arrowheads) larger than 3 mm containing low-level internal echoes

There is no consensus on the threshold values used to define varicocele by the maximum venous diameter. A diameter of 3 mm or more is commonly considered diagnostic for varicocele (Fig. 1), but lower and higher cut-off values have been reported. Cina et al. found an upper value, defined as the 97th percentile, of 3.7–3.8 mm, and showed that the mean diameter is different depending on the measurement site: 2.62 ± 0.53 mm in the spermatic cord, and 2.33 ± 0.56 mm in the peritesticular veins [63]. Karami et al. measured the diameter of the largest testicular vein at four different sites in both upright and supine positions, with or without the Valsalva manoeuvre [61]. They concluded that the best technique for examining patients with suspected varicocele is in the upright position during the Valsalva manoeuvre, and the best site for venous diameter measurement is at the level of epididymal head. Using these parameters, the best size threshold for differentiating normal from clinical varicoceles was 2.65 mm, with sensitivity and specificity of 91% and 89%, respectively. Metin et al. reported that a venous diameter of > 2.95 mm during Valsalva was associated with a sensitivity and specificity of 84% for the diagnosis of clinical varicocele [64]. Half of the patients with spermatic vein diameters of 3–4 mm, and all patients with spermatic vein diameters of 5–6 mm had palpable varicoceles. Eskew et al. reported an accuracy of 63% using cut-off values of 3.6 mm and 2.7 mm for clinical and subclinical varicoceles, respectively [57]. The optimal cut-off value for Pilatz et al. was 2.45 mm, with sensitivity and specificity of 84% and 81%, respectively [58]. Chiou et al. developed a more complex scoring system for varicocele which considers the maximum vein diameter and the sum of the diameters of up to six veins of the dilated plexus [46].

Given the existing wide methodological variability, it is evident that standardization is needed to obtain reproducible results in future studies. For measurement of all quantitative parameters, including venous diameter, it is critically important to document the patient’s position and define the sampling site unequivocally. Non-evidence-based recommendations can be offered advising how the study should be performed. Reviewing the literature, the majority of investigators examine the patient in both the supine and upright positions during the Valsalva manoeuvre, and accept a size threshold of 3 mm during the Valsalva manoeuvre as diagnostic for varicocele [45, 56–59]. The largest vein is measured in the majority of cases, irrespective of its location [53–55, 57, 58, 60, 62]. It is, therefore, advisable to perform US in this way.

When measuring venous diameter, it is critically important to indicate the patient’s position, measurement site (relative to the testis or to the cord), and whether the measure was obtained during the Valsalva manoeuvre or at rest. The ESUR-SPIWG recommends measuring the largest vein, irrespective of location, while standing and during the Valsalva manoeuvre. When the venous diameter is measured in this way, 3 mm or more is considered diagnostic for a varicocele (Table 2, recommendations 2–4).

## Systematic review to address question 4

Another debated point is whether measurement of testicular volume is needed and, if so, how it should be performed. Over 80% of the testicular volume is made up of seminiferous tubules and germ cells [65]. Therefore, reduction in germ-cell number results in a smaller volume. Unfortunately, the pooled data for normal testicular volume measured at US vary widely, ranging between 8 and 18 ml in the adult population. In part, this may be due to variation in measurements between US and different imaging modalities [66] and variation in individual practice but, more likely, it is explained by inaccuracies in US measurement and differences in how measurements are obtained. Evaluation of testicular diameters should be as accurate as possible to obtain a good estimate of the testicular volume at US, and the technique needs to be standardized. Compression should be avoided,the testis is very sensitive to probe compression, which significantly influences diameter measurements.

US assessed testicular volume is obtained from the three linear diameters of the testes—length (L), width (W) and height (H), and varies according to the mathematical formula applied for calculation. Three different formulas are used in the literature. The most common is the ellipsoid formula, which is also the one most widely used for automated volume calculation by US systems. Using this formula, the volume is obtained by multiplying the product of the three testicular diameters by 0.52 (*V* = L × W × H × 0.52) [67]. In another commonly used formula, width and height of the testis are considered to be equal, and testis volume is calculated as a prolate ellipsoid (*V* = L × 2W × 0.52). A third formula, introduced by Lambert et al., multiplies the product of the three diameters by 0.71 (*V* = L × W × H × 0.71) [68]. Mbaeri et al. compared the measurements of testicular volume using the three formulas in 62 patients undergoing therapeutic bilateral orchidectomy [69]. The gold-standard volume was calculated by water displacement of the orchidectomy specimen. The results showed that all three methods underestimated testicular volume,the best of the three was Lambert’s formula. A better performance of Lambert’s formula was also demonstrated by Sakamoto et al. [70].

There are no uniform reference values for the normal testicular volume in different populations, probably because measurements obtained in different studies are biased by a variety of circumstances such as the geographic areas and other environmental factors, nutritional status, and ethnicity [71–73]. At US, a total testicular volume of 20–24 ml or more is indicative of normal testicular function for Caucasians and Africans [65, 66, 74–76], while Asians are reported by some investigators as having slightly smaller testes [65, 77]. Bahk et al., however, did not confirm this difference [73].

Studies in infertile men have shown a direct correlation between semen parameters, sex hormone levels and testicular volume measured at US [76]. An average US-detected testicular volume in infertile patients ranges from 10 ml [78] to 13 ± 5 ml [70]. Comparing 136 infertile men and 100 fertile controls, Tijani et al. reported significantly smaller single testis volumes in infertile patients (15.32 ± 3.1 ml in infertile vs. 19.89 ± 3.8 ml in the control group) [74].

In patients with idiopathic hypogonadotropic hypogonadism and micropenis, Sun-Ouck et al. and Rajendra et al. found a significant increase of the testicular volume after medical treatment [79, 80].

A relationship between the testicular volume, measured at US, and varicocele has been documented. Zampieri et al. reported that venous reflux is associated with testicular atrophy and varicocele repair is associated with testicular volume increase [81–83].

The ESUR-SPIWG recommends measuring testicular volume in all patients with varicoceles as a surrogate evaluation of testicular function. The formula used to calculate the volume from the three diameters should be reported. Lambert’s formula is preferred (Table 2, recommendations 5–6).

## Systematic review to address question 5

The ESUR-SPIWG acknowledges that a standardized US examination is required for varicocele evaluation in which grey scale, colour Doppler US and spectral Doppler analysis are performed bilaterally with and without Valsalva, while standing and supine (Table 2, recommendation 7).

The ESUR-SPIWG recommends a complete grey scale and colour Doppler investigation with spectral analysis of flow signals in the dilated veins. Furthermore, all parameters should be assessed bilaterally with the patient in both the supine and upright positions. There is no evidence that this approach is necessary in all cases in clinical practice, but it helps to improve standardization and assists comparison between different studies. It also provides harmonization of technique among different centres and improves reproducibility of results. In general, the upright position is more informative and, for standardization purposes, is preferred for measurement of vein diameters and Doppler analysis.

The first part of the examination is grey-scale US. High-resolution images are obtained. The testes are measured with the patient lying supine, and the presence of enlarged (> 3 mm) venous structures demonstrated. The patient is then placed in the upright position and the largest vein (irrespective of the location) is identified and measured during the Valsalva manoeuvre. The second part of the study is evaluation of reflux. The equipment is set to detect slow flow. The pulse repetition frequency is minimized, wall filter set to minimum and gain to the maximum below noise threshold; this can be obtained by increasing the gain up to the level at which artefacts are visible and then decreasing it to a level at which they just disappear.

Colour Doppler and spectral analysis should be performed and interrogation should be obtained at the level of the inguinal canal, in the supratesticular region and in vessels at the level of the testis.

## Systematic review to address question 6

The ESUR-SPIWG recommendations emphasise that reflux evaluation is the most important component in the Doppler US examination of varicoceles. Therefore, spectral analysis should supplement colour Doppler interrogation. The essential parameter to measure is the duration of reflux; measuring the reflux peak velocity is not necessary (Table 2, recommendation 8–9). In patients with varicoceles, reflux is considered to be the primary pathologic process that causes testicular damage [84]. Although the precise mechanism through which varicoceles can affect spermatogenesis remains elusive [1], it is thought that if reflux is eliminated, the negative effect on spermatogenesis could reverse [85]. This is at the basis of therapeutic strategies for varicocele correction, whose goal is to improve sperm characteristics by removing retrograde flow within the internal spermatic veins [39, 86–88].

Several investigations support this scenario, showing that Doppler evaluation of reflux is critical for the diagnosis of varicoceles, helps predict the probability of postoperative semen improvement, and has a role in determining the therapeutic approach. It has been shown, in particular, that after varicocele repair, the best postoperative improvement of semen quality is obtained for patients with preoperative evidence of significant reflux at Doppler interrogation. Continuous basal reflux or reversal of flow with Valsalva manoeuvre before varicocele repair is strongly associated with improvement in sperm count and motility on postoperative semen analysis [41, 60, 84].

**Fig.2:**
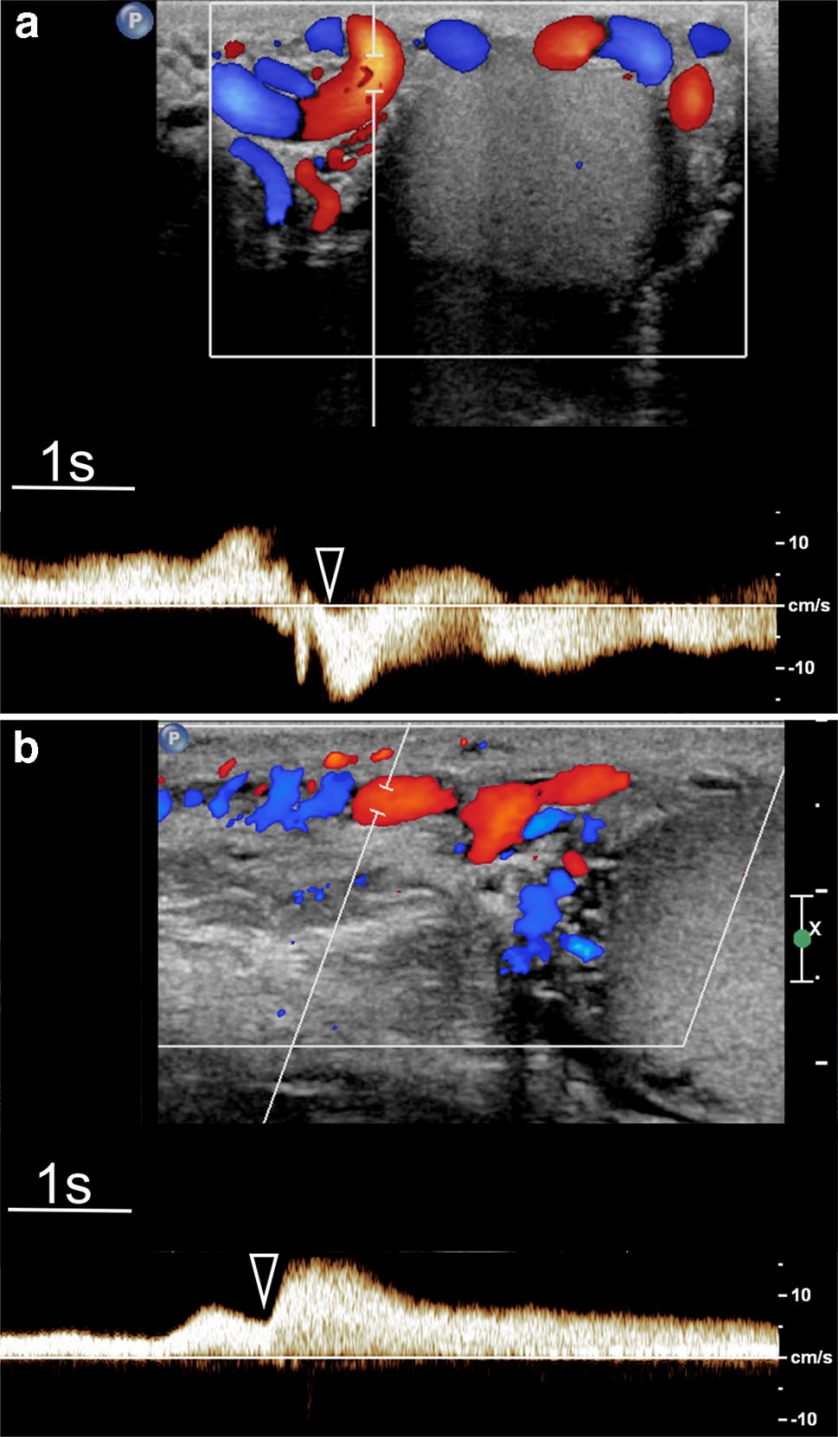
Spectral Doppler analysis in varicocele. Changes of reflux while standing during Valsalva (arrowhead). **a** Flow inversion. **b** Flow increase showing a plateau throughout Valsalva. In both cases reflux persists for more than 2 s

Diagnosis of reflux is obtained by combining colour Doppler interrogation and spectral analysis. The first analysis is with the colour Doppler mode, which provides a panoramic view of the spermatic vessels, their relationship with testis, information in real time about direction of flow and on how it changes in different positions and during the Valsalva manoeuvre. Use of high-end equipment with good colour Doppler sensitivity is recommended to avoid false-negative results for varicocele detection. Using high-specification equipment, reflux can often also be appreciated at grey-scale US. The examination technique is crucial. Reflux can be missed with the patient supine and it is best detected in the standing position during the Valsalva manoeuvre [62]. Colour Doppler interrogation is subjective, and findings must be substantiated with spectral Doppler analysis which allows measurement of the duration and characteristics of reflux (Fig. 2; this should be quantified [51]. Measurement of reflux peak velocity is optional and can be problematic because it requires a careful angle correction to be obtained in vessels with tortuous courses.

## Systematic review to address question 7

When imaging varicoceles, a key point to be clarified is how long the reflux should last to make the diagnosis. Different classification systems use different duration thresholds for diagnosis and grading of reflux [43, 47–51, 63, 89, 90].

In their pioneer study in 1989, Dhabuwala et al. investigated the duration of reflux with dual-frequency bidirectional Doppler [43]. Varicocele was classified as mild if reflux lasted less than 2 s, and severe if it lasted more than 2 s. Cornud et al. also classified varicoceles according to the duration of reflux: described as brief (less than 1 s), intermediate (1–2 s) and permanent (> 2 s) [47]. Brief reflux is considered as physiological in this study and by other investigators [50, 51]. Permanent reflux (> 2 s) was not palpable in 40% of the patients but was always evident at venography. After varicocele repair, reflux disappeared in 70% of cases and changed from continuous to intermediate type in 20% [47]. Oyen et al. also considered a cut-off of 2 s diagnostic for the diagnosis of a varicocele [48]. Only one study on 145 healthy, asymptomatic subjects with normal clinical examination and semen analysis fixed the upper limit for physiological reflux to 3 s, with a velocity of 0.1 m/s [63]. The ESUR-SPIWG suggests a threshold of > 2 s for diagnosis of varicoceles, measured while standing and during Valsalva (Table 2, recommendation 10).

## Systematic review to address question 8

Several investigations suggest measuring the reflux peak velocity as a potentially useful Doppler parameter to predict the need for varicocele repair [63, 91–98]. Unfortunately, there is a lack of consensus on where and how to perform the measurement. In most of studies, the peak velocity is measured with the patient supine during the Valsalva manoeuvre [63, 91, 96, 98, 99]. In one the peak, velocity was measured upright with the patient breathing normally [95]. In another, the position of the patient is not reported [93]. Measurement were performed either in the largest vein [91, 98], in the spermatic cord just cephalad to testis [99] or, in general terms, where reflux was identified [63]. Three investigations do not report the measurement site [93, 95]. In most studies, angle correction was not performed and, therefore, the velocity values obtained cannot be regarded as being accurate [91, 95–99].

These differences are relevant, as flow velocity changes depend on the site of measurement [63], patient position and Valsalva [92]. Also, angle correction is essential in all Doppler velocity measurements.

Since the pooled data are inconsistent and biased by relevant methodological shortcomings, the ESUR-SPIWG does not recommended evaluation of reflux peak velocity in routine clinical practice (Tab.2, recommendation 11).

## Systematic review to address question 9

**Fig.3:**
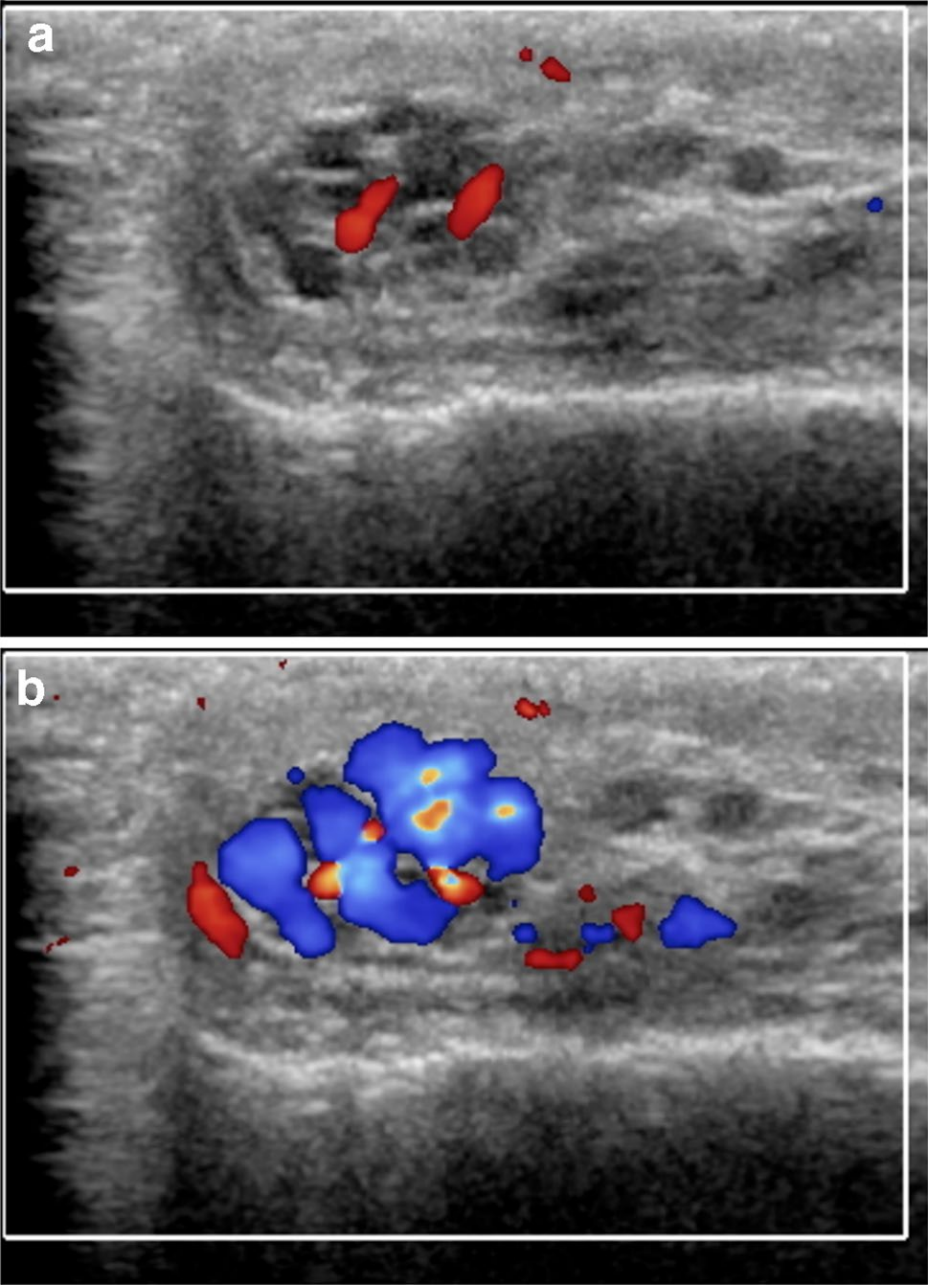
Sarteschi’s grade I varicocele. Colour Doppler images obtained at rest (**a**) and during Valsalva (**b**) showing dilated veins of the spermatic cord with reflux during Valsalva at the inguinal canal

**Fig.4:**
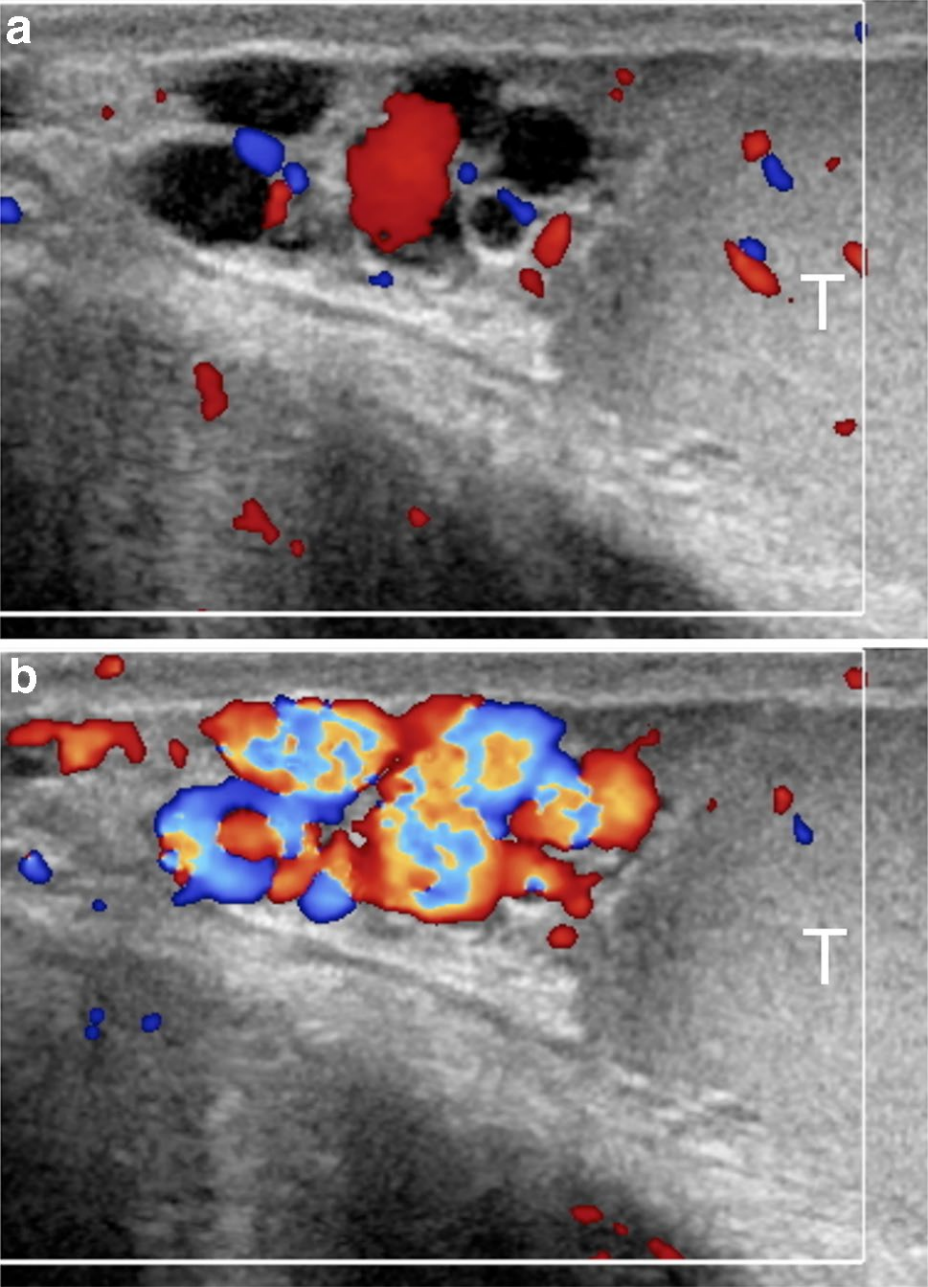
Sarteschi’s grade II varicocele. Colour Doppler images obtained at rest (**a**) and during Valsalva (**b**) showing dilated veins in the supratesticular region with reflux during Valsalva (T = testis)

**Fig.5:**
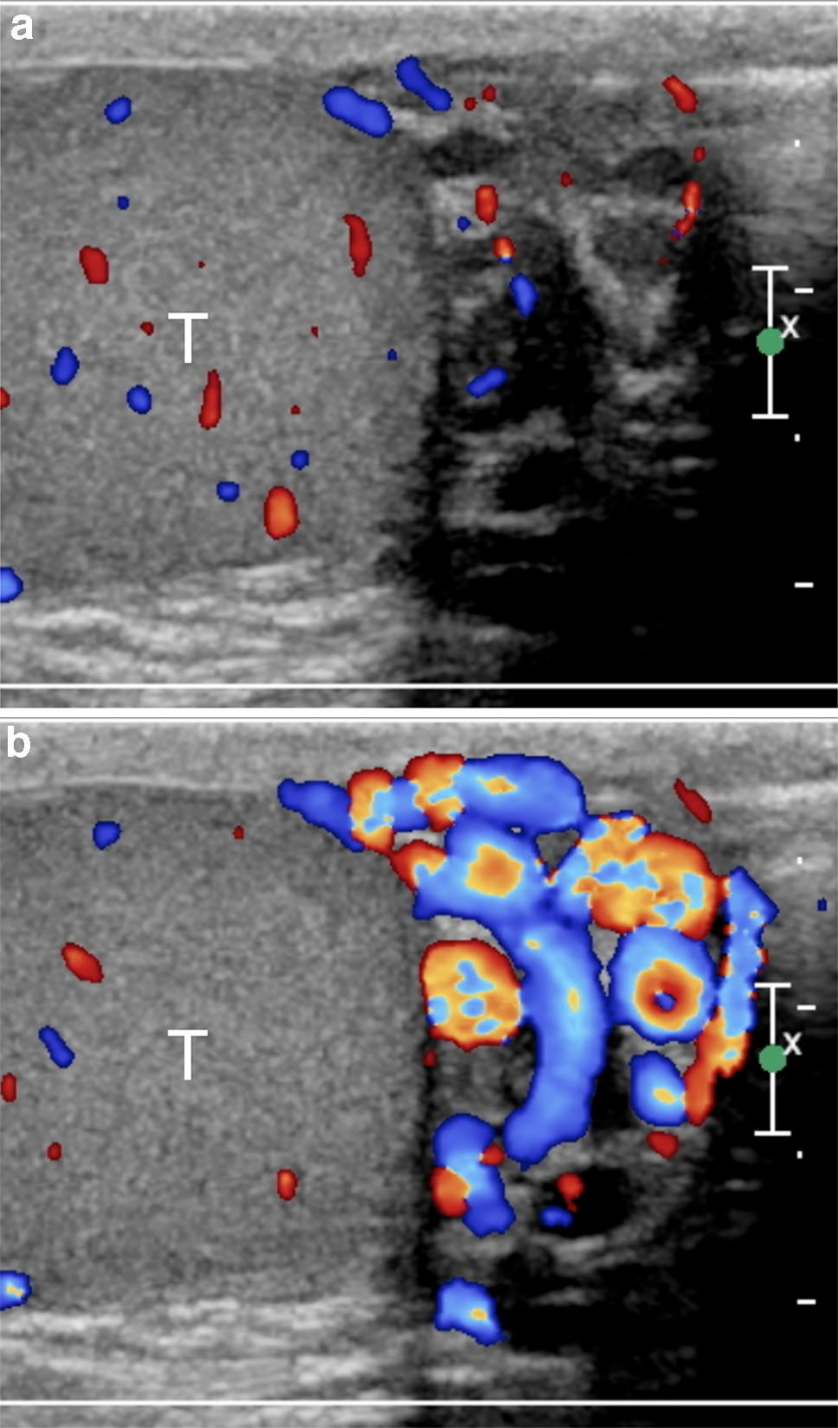
Sarteschi’s grade III varicocele. Colour Doppler images obtained at rest (**a**) and during Valsalva (**b**) showing dilated veins to the inferior pole of the testis (T) with reflux during Valsalva

**Fig.6:**
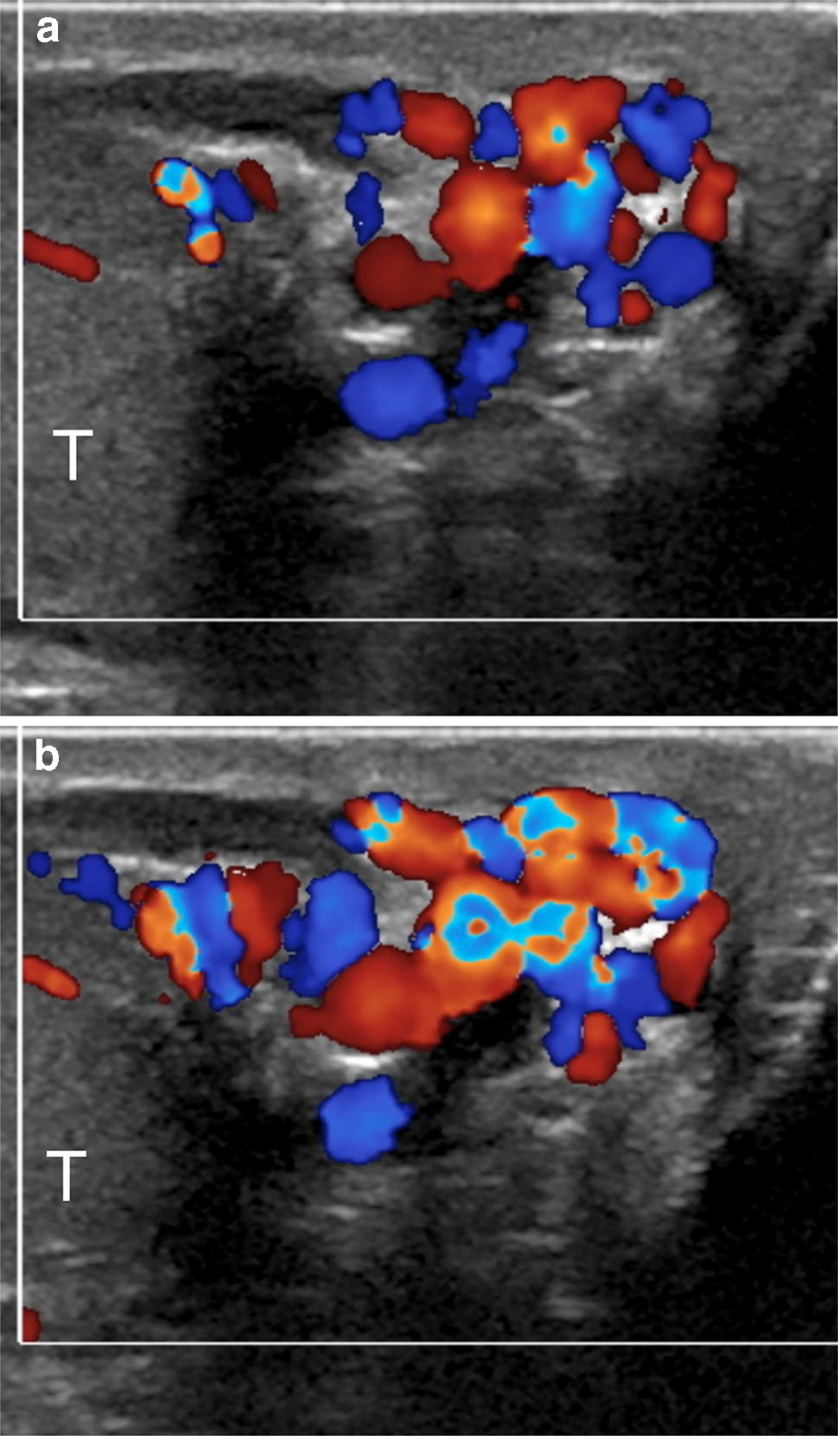
Sarteschi’s grade IV varicocele. Colour Doppler images obtained in supine position at rest (**a**) and while standing during Valsalva (**b**). Dilated veins with reflux are visible also at rest. Reflux increases while standing during Valsalva (T = testis)

One of the problems encountered when comparing different US studies for varicoceles concerns the variability of reporting. A standard report should be useful, in which all the relevant data are collected. The variable clinical practice in reporting patients with varicoceles reflects the differences in examination techniques. The ESUR-SPIWG recommends describing the examination technique and reporting all the US parameters used to evaluate the patient. It also suggests grading varicoceles according to the Sarteschi’s classification (Figs. 3, 4, 5, 6, Table 2, recommendations 12–13).

The following information should be included in the US report:

- Testicular volume, echogenicity and echotexture
- Presence of incidental testicular or extratesticular abnormalities other than varicoceles
- Presence of varices at grey-scale and colour Doppler US, and relationships to the testis (inguinal canal, supra-testicular, around the testis, intratesticular)
- Diameter of the largest vein (irrespective of the location) measured while standing and during the Valsalva manoeuvre
- Changes of flow at colour Doppler interrogation and spectral analysis in the spermatic veins according to the patient’s position and before and during Valsalva (length of reflux and changes in waveform characteristics while standing and during the Valsalva manoeuvre).

## Systematic review to address question 10

Several studies have investigated intratesticular Doppler waveforms to predict parenchymal damage in infertile patients with varicoceles [100–105]. As discussed in previous paragraphs, the mechanism underlying testicular damage in patients with varicoceles remains uncertain. Defective energy metabolism at the mitochondrial level following decrease of arterial inflow was proposed as one of the factors responsible for impaired spermatogenesis [106]. Pathophysiologically, it is hypothesized that impaired venous drainage causes an increase in venous pressure within the spermatic veins [100]. Venous stasis may also decrease the arterial blood supply and microperfusion of the testes resulting in hypoxia and, in turn, impaired testicular microcirculation [100].

Several studies have investigated whether changes in testicular vascularization were appreciable, since they could represent (indirect) confirmation of this theory. The purpose of these investigations was to provide insight into the pathophysiology of varicoceles, not to identify new parameters for clinical use in patient management. Earlier studies failed to demonstrate changes in testicular blood flow in varicocele patients [101, 102], a negative result confirmed also by a more recent investigation [107]. Other conflicting studies, however, did identify changes in flow Tarhan et al. measured arterial blood flow in the testicular artery of 62 infertile patients with varicoceles and 42 normal fertile subjects used as control group. They found ipsilateral decreased testicular arterial volume blood flow in patients with varicoceles compared to control patients reflecting, in the author’s opinion, impaired microcirculation [103].

Calculation of testicular volume blood flow, however, requires estimating the cross-sectional area of the testicular artery, a difficult evaluation in a millimetric vessel. Other authors used peak systolic velocity (PSV) and resistive index (RI) or pulsatility index (PI) as more reproducible and easily obtained parameters to estimate indirectly perfusion abnormalities. Biagiotti et al. found higher PSV and RI in the testicular arteries of patients with varicoceles [104]. Ünsal et al. reported similar results—interrogating the capsular arteries, they found significantly higher RI and PI values in patients with varicoceles, compared to controls [105]. These research studies are useful to provide an insight into the mechanisms that create testicular parenchymal damage in varicoceles. At present, however, there is insufficient evidence to recommend evaluation of intratesticular arterial Doppler waveforms for clinical use (Table 2, recommendation 14)*.*

## Systematic review to address question 11

There is a widely held belief that a unilateral right-sided varicocele should prompt investigation for a retroperitoneal process causing obstruction of the right internal spermatic vein. While this may be justified if a varicocele appears suddenly, be it right left, or bilateral, in the majority of cases the literature does not support this approach.

With an incidence of about 10% of varicoceles, bilateral clinical varicocele is relatively uncommon [108]. However, the incidence of subclinical right-sided varicocele is much more frequent. The World Health Organization reported that approximately 90% of right-sided varicoceles are undiagnosed at palpation [109], a fact confirmed by other investigations [110–113].

As clinical diagnosis is neither sensitive nor accurate, a right-sided varicocele is, in the majority of cases, identified at colour Doppler US when a study is requested to evaluate a clinically evident left-sided one [110–113].

Several authors consider varicocele a bilateral disease, although on the right side, the condition is often subclinical. This explains, in their opinion, bilateral testicular dysfunction in patients with unilateral left clinical varicocele [39, 112, 114]. Patients with left-sided varicocele, either clinical or subclinical, should, therefore, be examined carefully for bilateral varicoceles, and bilateral repair should be considered if a right subclinical varicocele is found [112, 114–117].

In adults, there is some evidence that simultaneous repair of left clinical and right subclinical varicocele is beneficial, as even a small subclinical unrepaired varicocele continues to have a detrimental effect on testis function [117–120]. Contrary opinions, however, exist [121]. In children and adolescents, the value of bilateral repair is even more debatable [94].

Isolated right-sided varicocele is found in less than 1% of patients [108]. In the majority of cases, it is subclinical, identified only during the Valsalva manoeuvre [111, 113]. Literature is scarce and is rarely focused on this topic [122].

A variety of causes can result in right-sided clinical varicocele, including impaired venous drainage from the testis by venous thrombosis, tumour invasion or compression. Isolated and/or palpable right-sided varicocele may occur in otherwise healthy patients with venous anatomical variations [110], such as in situs viscerum inversus [123].

Based on these considerations, the ESUR-SPIWG recommends undertaking colour Doppler US on both sides in patients with left-sided varicoceles, as a subclinical right-sided varicocele will be often revealed. In patients with an isolated clinical right-sided varicocele, it also recommends extending the US examination to the abdomen, to look for congenital anomalies and abdominal/retroperitoneal pathology (Table 2, recommendation 15–16).

## Systematic review to address question 12

The need for follow-up in patients with subclinical varicoceles is debated. Current literature supports the thesis that a varicocele has a detrimental effect on testicular size and function from childhood (prepuberal boys) and adolescence (Tanner stage *V* males from puberty to the age of majority) onwards and that this adverse effect increases with the grade of the varicocele [122]. The need for diagnosing, treating and/or following-up subclinical varicoceles, however, still remains controversial, especially in prepuberal boys. Current evidence shows that varicocele repair significantly improves semen parameters in men with clinical varicoceles [32, 34], but not when the varicocele is subclinical [124]. Also, there is no proven benefit in treating men with a varicocele and normal semen parameters [125].

In adolescents with varicoceles, there is a significant risk of over-treatment since most of them will have no problem achieving pregnancy later in life [126].

There is further evidence that subclinical varicoceles can progress to clinically evident disease. In a paediatric population, Cervellione et al. found progression in 28% of patients during a 4-year period [127]. Zampieri et al. showed a significantly higher progression rate in adolescent athletes, compared to the general population [128].

Testicular atrophy associated with varicoceles is currently the most common indication for prophylactic varicocele repair in adolescents [19, 129], but a study suggests a 6–12 month observation period prior to surgery, or prolonged follow-up if patients present with normal semen analysis at diagnosis and agree to annual semen analysis and scrotal US [130].

As surgical repair is not indicated for all patients with varicoceles, and disease progression may occur, follow-up is advised for non-operated patients. In view of the increased risk for progressive testicular dysfunction in adolescent males compared to older patients, there are two groups of patients with varicoceles in which follow-up is specifically recommended: non-operated adolescents with testicular atrophy, and adolescents and young adults (young males who have attained the age of majority) presenting with subclinical varicoceles and both normal testicle volume and semen analysis. In these patients, annual follow-up is advised by means of physical examination, scrotal US including measurements of testis size and, if patients are willing to comply, semen analysis [130, 131].

Based on the currently available literature, the ESUR-SPIWG recommends follow-up of subclinical varicoceles for untreated adolescents and for young adults with normal testicular volume and normal semen analysis (Table 2, recommendation 17).

## Systematic review to address question 13

Another debated point is whether to undertake US follow-up after varicocele repair. The ESUR-SPIWG recommends US follow-up to identify early postoperative complications, and later if semen analysis remains unsatisfactory (Table 2, recommendation 18–20).

In addition to the risks of infection, bleeding, and delayed wound healing, as for all surgical procedures, recurrence of the varicocele and hydrocele formation are the most common complications following varicocele surgical repair.

When a venous embolization technique is employed, the main reported complications are coil migration, vascular trauma, thrombophlebitis of the pampiniform plexus and contrast agent reactions. Technical success rate is reported as being equivalent to that of surgical techniques [132].

With the exception of an early study in which colour Doppler US was not found effective in assessing the outcome of varicocele repair [133], there is agreement that US can be used early after treatment in case of postoperative complications, and later, when needed, to evaluate morphology of the pampiniform plexus, testicular volume and signs of persistent or recurrent disease [133–147]. Clinical evaluation after surgery, especially in high-grade varicocele, usually still detects enlarged veins, but only colour Doppler with spectral analysis can discriminate if there is persisting venous reflux. Hence, colour Doppler US is important in detecting persistent reflux or recurrence.

The clinical workup of patients differs as regards indications for imaging following varicocele repair, particularly timing, and length of sonographic follow-up. No clinically and scientifically based recommendations are available on this specific clinical problem.

In several centres, US is considered after varicocele repair only if early or late complications appear (such as hydrocele, pain, epididymitis), if there is evidence of recurrence on clinical examination or if postoperative sperm analysis is unsatisfactory. In other centres both sperm analysis and US are performed 3 months after varicocele repair. In other centres sperm analysis and US are performed 3 months and 1 year after varicocele repair, with the rationale that recurrent varicocele may only appear one year or later after the procedure [39, 134, 138].

If varicocele repair is performed for treatment of subfertility, then sperm analysis must form the basis of follow-up. If semen analysis improves, scanning is usually unhelpful for the patient, while if semen analysis remains unsatisfactory, US can be considered to look for persistent reflux or recurrent disease.

## Systematic review to address question 14

It is commonly thought that US should be extended to the abdomen in newly discovered varicoceles to identify tumours. This practice, however, is not fully supported by the available literature. The classic triad of symptoms of renal cell carcinoma (RCC) is haematuria, flank pain, and a palpable abdominal mass. Other paraneoplastic signs such as fever or general malaise may also be present with retroperitoneal tumours. A number of case reports and small case series suggest that varicoceles are a possible mode of presentation of retroperitoneal tumours [148–152]. This led to the dogma that an extended abdominal ultrasound examination should be performed in all patients with a newly diagnosed varicocele. Varicocele is, however, very common, whereas retroperitoneal tumours are rare. The incidence of renal tumours is 13.4 per 100,000 per year, and that of retroperitoneal tumours is 0.3 per 100,000 per year in the male population [148]. There is no data to suggest that finding a retroperitoneal or renal tumour as a cause for a varicocele is any more likely than discovering an incidental tumour in a male patient without a varicocele, or in any other patient group. In a much-quoted series detailing the clinical manifestations of renal cell carcinoma with emphasis on the presence of varicoceles [149, 151, 153], the prevalence of co-existing retroperitoneal tumours and a varicocele is low (incidence of 2.8–3.3%) [149, 151]. Moreover, varicocele, whether acute, symptomatic or an incidental finding, is almost never a sole feature of a renal or retroperitoneal tumour and some other features of the tumour are usually evident from the history or examination. Ding et al. reported a retrospective analysis which included 1028 patients with pathologically confirmed unilateral RCC [154]. In 333 patients, paraneoplastic signs were present as the initial symptoms comprising pyrexia in 175 cases (52.6%), anaemia in 146 cases (43.8%), hypertension in 101 cases (30.3%), and varicocele in 12 cases (3.6%). In this study, varicocele was found to be related significantly to advanced cancer stage and therefore other clinical signs (abdominal mass, flank pain) and paraneoplastic signs more likely co-existed at presentation. Conversely, Pauruso et al. found no abdominal tumours at US in all their patients examined for varicoceles [49].

The mechanism for the development of a symptomatic varicocele is not always extension of tumour/thrombus along the renal vein. Pathological extrinsic compression of the venous drainage of the pampiniform plexus by the primary tumour or enlarged lymph nodes, or an increased blood supply to the tumour may also lead to a local increase in venous pressure, or an increase of reflux of blood into collaterals [155].

Besides advanced retroperitoneal tumours, a variety of non-tumour lesions have been associated with secondary varicoceles including non-neoplastic enlarged lymph nodes, non-neoplastic renal vein thrombosis, large aneurysmal dilatations, pancreatic pseudocysts, renal arteriovenous malformations, and the nutcracker (left renal vein entrapment) syndrome [156–158].

Anecdotally, a secondary varicocele is often considered when the disease is found solely on the right side. Isolated right-side varicocele occurs in a minority of patients with clinical varicoceles [110, 115]. Tumour thrombus in the right renal vein seldom results in the development of a right-sided varicocele, because the right spermatic vein commonly drains into the inferior vena cava, with the incidence of the right spermatic vein draining into the right renal vein reported in less than 5% of individuals [159–161]. Moreover, it has been shown that a secondary varicocele is as likely on the left side as the right, therefore, considering a secondary varicocele only in the rare situation in which it develops on the right is misleading [162].

There are no data that currently support extended US examination to the abdomen in all patients with a newly diagnosed varicocele. An exception to this is in children less than 9 years of age presenting with a varicocele. Epidemiological studies have demonstrated that varicoceles develop at puberty. Oster et al. observed that no varicoceles were detected in 188 boys 6–9 years of age [163]. Akbay et al. evaluated the prevalence of varicoceles in 4052 boys aged 2–19 [164]. They reported that the prevalence of varicoceles was < 1% in boys aged 2–10. In the child less than 9 years of age, where acute varicoceles are normally not seen, a renal or retroperitoneal tumour is a possibility and should be excluded [165].

In conclusion, suspicion of a secondary varicocele can arise when a clinically evident varicocele presents acutely, and remains tense in a supine position. Secondary subclinical varicocele has not been reported. The ESUR-SPIWG believes that US examination should always be extended to the abdomen in children less than 9 years presenting with an acute varicocele, to rule-out a renal or retroperitoneal tumour. In adults, the alleged association between varicoceles and retroperitoneal masses is based on a limited number of series and case reports in which the varicocele was a late sign of a symptomatic, palpable lesion [152, 166–169].

**Fig.7:**
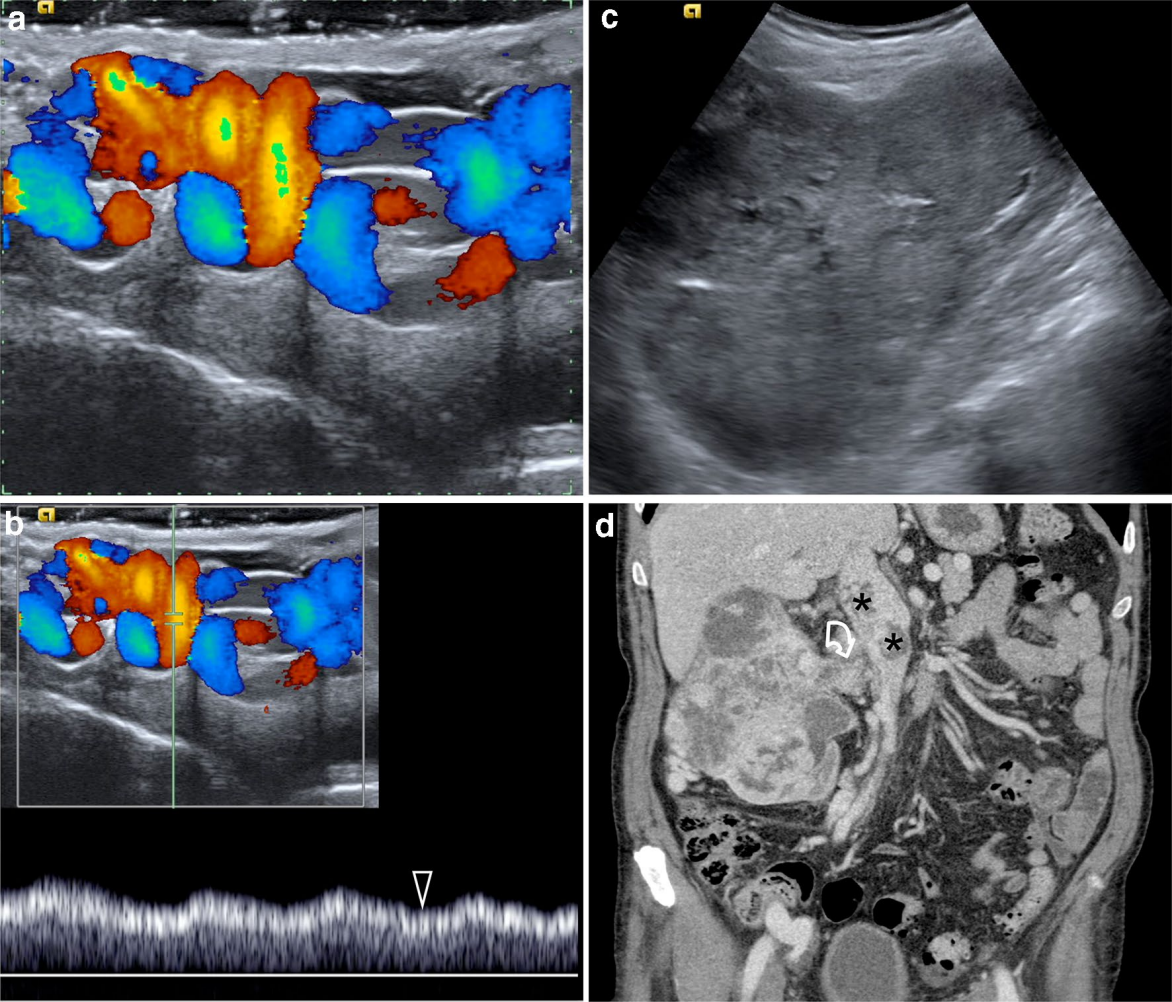
Secondary varicocele in an 85-year-old patient presenting with a right scrotal lump. **a** Colour Doppler ultrasound of the right spermatic cord shows markedly dilated pampiniform plexus with basal reflux. **b** Spectral Doppler interrogation obtained in an upright position shows no changes during Valsalva’s maneuver (arrowhead). **c** Ultrasound interrogation of the right kidney shows a large renal tumour. **d** Contrast-CT scanning showing a large right renal tumour growing into the right renal vein (curved arrow) and inferior vena cava (asterisks)

Although the possibility of missing an undetected retroperitoneal lesion during a Doppler investigation for varicocele when the abdomen is not evaluated in an adult is very unlikely, it cannot be completely excluded (Fig. 7). Patients often undergo US examinations without a full clinical evaluation, and even obvious abdominal findings can be missed. As a consequence, the ESUR-SPIWG believes that the sonologist is fully justified in extending the US examination to the abdomen when the varicocele is of acute onset, whatever the side (left or right), and particularly when it remains unchanged in the supine position. Otherwise, the US practitioner should refer the patient to an internal medicine specialist for a complete diagnostic evaluation including an abdominal examination (Table 2, recommendation 21–22)*.*

## Systematic review to address question 15

The ESUR-SPIWG recommend considering other pathologies when performing US in patients with clinical diagnosis of varicoceles (Table 2, recommendation 23)*.* Not all tubular extratesticular structures, either palpated or identified at US, are varicoceles. Colour Doppler interrogation is important to differentiate between varicocele and other tubular structures on grey-scale US, such as spermatoceles and cyst clusters, tubular ectasia and postvasectomy changes.

Patients presenting with uncommon conditions [170, 171], as well as patients with extratesticular cavernous haemangiomas, lymphangiomas, and arteriovenous malformations can present with a non-specific diagnosis, or with suspicion of a varicocele based on their clinical presentation [172–174]. Although MRI is the modality of choice for delineating the extent of these abnormalities, in clinical practice, the diagnosis is usually obtained with US. On US, cavernous haemangiomas present with a heterogeneous echo texture and increased through transmission, showing septa and enlarged vascular spaces. Phleboliths may be seen as foci with distal acoustic shadowing. The tortuous vessels inside the lesion may mimic a varicocele on grey-scale US. At Doppler interrogation, flow velocity is often too slow to be detected, even with the patient standing and during the Valsalva manoeuvre. MRI shows intermediate-to-low signal intensity on T1-weighted images, and very high signal intensity on T2-weighted images. The lesion and serpiginous vessels show enhancement after contrast medium administration. Focal signal voids may be seen, consistent with thrombi [172, 173]. Lymphangiomas are composed of dilated lymphatic channels,they may present as lobulated cystic masses or with findings similar to those of haemangiomas. The dilated lymphatic channels of lymphangiomas do not enhance after contrast medium injection. Lymphangiomas present on MRI as lobulated scrotal masses with intermediate-to-low signal intensity on T1-weighted images and high signal intensity on T2-weighted images [172]. Arteriovenous malformations are characterized by abnormal connections between arteries and veins. At colour Doppler and duplex Doppler imaging, they show prominent vessels with low-resistance arterial flow signal and high-velocity venous components. These features allow differentiation from varicoceles, in which only venous flow are recorded [174]. Arteriovenous malformations appear at MRI as a tangle of abnormal vessels, frequently with internal flow voids on both T1- and T2-weighted images produced by high-velocity flow [175].

Zinner syndrome has also been described as a mimic for varicocele. It is a rare developmental anomaly of the mesonephric duct consisting of unilateral renal agenesis, ipsilateral seminal vesicle cyst, and ipsilateral ejaculatory duct obstruction resulting in dilatation of the epididymis and vas deferens. In Zinner syndrome, the dilated vas deferens and epididymis can simulate venous dilatation. Reflux can be artefactually recorded at colour Doppler interrogation, due to sperm movement during the Valsalva manoeuvre [176].

A few uncommon pathological conditions can mimic intratesticular varicocele [177]. The Valsalva manoeuvre can be very important to differentiate between intratesticular varicocele adjacent to the mediastinum testis and tubular ectasia of the rete testis. Intratesticular arteriovenous malformations and intratesticular haemangiomas can be similar morphologically to intratesticular varicocele, but present with arterial flows and an arterialized-venous spectral waveform.

## Conclusion

Guidelines have increasingly become a familiar part of clinical practice. They can improve the quality and consistency of care, promoting use of procedures of proved benefit and discouraging use of ineffective ones [178]. Imaging guidelines, in particular, have been developed to address specific and controversial problems on how to perform imaging procedures [179–182], and to offer explicit recommendations for operators who are uncertain about how to proceed [183–189]. They fix technical standards, and provide authoritative recommendations that reassure practitioners about the appropriateness of their work [190–192].

This systematic literature review of US imaging for varicocele shows significant variability. There is, however, good evidence to support the contention that an accurate imaging diagnosis of patients with varicoceles is important to guide clinicians in making effective treatment decisions. The recommendations and guidelines released by the ESUR-SPIWG are aimed to produce a framework that allows standardisation across future studies with the intention of clarifying the role of US in varicocele assessment.
